# Molecular dynamics studies unravel role of conserved residues responsible for movement of ions into active site of DHBPS

**DOI:** 10.1038/srep40452

**Published:** 2017-01-12

**Authors:** Ranajit Nivrutti Shinde, Subramanian Karthikeyan, Balvinder Singh

**Affiliations:** 1 CSIR-Institute of Microbial Technology, Council of Scientific and Industrial Research (CSIR), Sector 39-A, Chandigarh 160036, India

## Abstract

3,4-dihydroxy-2-butanone-4-phosphate synthase (DHBPS) catalyzes the conversion of D-ribulose 5-phosphate (Ru5P) to L-3,4-dihydroxy-2-butanone-4-phosphate in the presence of Mg^2+^. Although crystal structures of DHBPS in complex with Ru5P and non-catalytic metal ions have been reported, structure with Ru5P along with Mg^2+^ is still elusive. Therefore, mechanistic role played by Mg^2+^ in the structure of DHBPS is poorly understood. In this study, molecular dynamics simulations of DHBPS-Ru5P complex along with Mg^2+^ have shown entry of Mg^2+^ from bulk solvent into active site. Presence of Mg^2+^ in active site has constrained conformations of Ru5P and has reduced flexibility of loop-2. Formation of hydrogen bonds among Thr-108 and residues - Gly-109, Val-110, Ser-111, and Asp-114 are found to be critical for entry of Mg^2+^ into active site. Subsequent *in silico* mutations of residues, Thr-108 and Asp-114 have substantiated the importance of these interactions. Loop-4 of one monomer is being proposed to act as a “lid” covering the active site of other monomer. Further, the conserved nature of residues taking part in the transfer of Mg^2+^ suggests the same mechanism being present in DHBPS of other microorganisms. Thus, this study provides insights into the functioning of DHBPS that can be used for the designing of inhibitors.

In bacteria, riboflavin (Vitamin B_2_) is produced via riboflavin biosynthesis pathway. Riboflavin is the universal precursor of flavocoenzymes - flavin mononucleotide (FMN) and flavin adenine dinucleotide (FAD). It has been estimated that up to 3.5% of bacterial proteins use flavocoenzymes^1^. These flavocoenzymes are involved in various biochemical reactions such as oxidation, epoxidation, sulfoxidation, amine oxidation, selenide oxidation, Baeyer–Villiger oxidation, phosphite ester oxidation, hydroxylation, halogenation, and dehydrogenation, which are part of different metabolic pathways such as the citric acid cycle, β-oxidation, degradation of amino acids, and detoxification of a vast spectrum of xenobiotics^2^. FMN and FAD are also involved in the biosynthesis of steroids, thyroxin, coenzyme A, coenzyme Q, heme, and pyridoxal 5′-phosphate^3^,^4^. Moreover, these are essential in numerous physiological processes such as light sensing^5^, light driven DNA repair^6^, bacterial bioluminescence^7^,^8^, and regulation of biological clock^9^. These roles of FMN and FAD make riboflavin as an essential element for microorganisms. The riboflavin biosynthesis pathway is present in plants and many pathogens. Interestingly, it is absent in animals, and they obtain riboflavin from the nutritional sources. This makes the riboflavin biosynthesis pathway a rich source of targets to design selective anti-infective agents. Additionally, it provides an alternate source of novel targets urgently needed to tackle the problem of antibiotic resistance^10^,^11^.

Seven enzymes take part in the bacterial riboflavin biosynthesis pathway. These are GTP cyclohydrolase II, pyrimidine deaminase, pyrimidine reductase, putative pyrimidine phosphatase, 3,4-dihydroxy-2-butanone-4-phosphate synthase (DHBPS), lumazine synthase, and riboflavin synthase. DHBPS converts the substrate D-ribulose 5-phosphate (Ru5P) into L-3,4-dihydroxy-2-butanone-4-phosphate (DHBP) and formate using Mg^2+^ as co-factor^4^. According to the proposed reaction mechanism, Mg^2+^ makes coordinate bonds with Ru5P, water, and enzyme residues, initiates reaction by proton abstraction, advances it through enolization, protonation, dehydration, and skeletal rearrangement to release the products, DHBP and formate^12^,^13^,^14^,^15^. The structures of DHBPS in its apo, Ru5P or Ru5P-ion bound form have been solved for many organisms viz. *Escherichia coli*^16^*, Magnaporthe grisea*^17^,^18^*, Methanocaldococcus jannaschii*^14^,^19^*, Yersinia pestis, Candida albicans*^20^,^21^*, Streptococcus pneumoniae*^22^*, Mycobacterium tuberculosis*^23^,^24^*, Salmonella typhimurium*^25^*, and Vibrio cholerae*^26^. In these structures, DHBPS mostly exists as homo-dimer (monomer-A and monomer-B), and Ru5P binds in the active site of each monomer. An active site cavity is formed mostly by loops of one monomer with a side being lined by loops of another monomer. Accordingly, in *Vibrio cholera* DHBPS, monomer-A active site is surrounded by loop-1 (34–41), loop-2 (82–98), and loop-3 (175–185) of the monomer-A, and loop-4 (103′–111′) and loop-5 (132′–138′) of the monomer-B^26^. A similar arrangement of loops is found in the active site of monomer-B, as shown in Supplementary Fig. S1. DHBPS-Ru5P structures have been solved for *C. albicans*^20^,^21^*, S. typhimurium*^25^, and *V. cholerae*^26^ whereas DHBPS-Ru5P-ion complexes are solved for *M. jannaschii*^14^ and *V. cholerae*^26^ in the presence of inactive ions such as Zn^2+^ and Ca^2+^. Numerous structural studies have speculated that inactive ions occupy the same position as that of active Mg^2+^, and mimics native like binding^14^,^18^,^25^,^26^. Failure of inactive ions to initiate the reaction, even if these bind at the same position, forming the same number of co-ordinate bonds, having similar charge and ionic radii to that of Mg^2+^, is attributed to their lower Lewis acid strength^14^.

Earlier, our group has solved the structures of *Vibrio cholerae* DHBPS in complex with Ru5P as well as in complex with Ru5P and Zn^2+ ^26. DHBPS-Ru5P-Zn^2+^ structure shows two Zn^2+^ along with Ru5P bound in each active site^26^. These ions occupy M1 and M2 positions forming coordinate bonds with the surrounding water, Ru5P, and DHBPS residues as shown in Supplementary Fig. S2. This assembly of the substrate-dimetal center is well established by several ion bound structures of DHBPS^14^,^18^,^25^,^26^. M1 and M2 positions of the ions coincide in all the structures of DHBPS that have complexed with metal ions. However, for *M. jannaschii* DHBPS^14^, the M2 position is slightly changed. Ca^2+^ at this position forms interactions with nearby ligands with longer bond lengths than Zn^2+^ at the M1. It has been speculated that the metal ion at the M1 position is sufficient to initiate the catalytic reaction, and the second ion may be involved in avoiding unproductive side reactions of highly reactive intermediates^14^. It has also been put forward that more DHBPS structures may validate the exact positions of two metal ions, especially in the presence of Mg^2+^ 26. Zn^2+^ bound structure of DHBPS-Ru5P complex is obtained by soaking the crystals of DHBPS-Ru5P complex into the solution of ZnCl_2_^26^. This experiment suggests that ions enter the active site from the bulk solvent. However, the mechanism behind entry and positioning of ions into the active site is still unknown. Comparison of DHBPS-Ru5P and DHBPS-Ru5P-Zn^2+^ structures show that the loop-2 is partially ordered in the absence of ions, but becomes fully ordered in the presence of Zn^2+^. A similar change is also observed in conformation of Ru5P where flexible Ru5P becomes more rigid in the presence of Zn^2+^. Overall, it reveals that the presence of ions brings noticeable changes in DHBPS-Ru5P complex.

So far, most of the crystallographic studies that have been performed in the presence of inactive metal ions display interactions presumably formed by the Mg^2+^. Despite the availability of the crystal structures of DHBPS, and its complexes with metal ions and the substrate, the molecular mechanistic insights such as reordering of loop-2 in the presence of the metal ions, events marking the entry of metal ions, subsequent conformational changes in substrate (Ru5P) etc. are still elusive. Therefore, molecular dynamics (MD) simulations of DHBPS-Ru5P complex have been carried out in the presence of Mg^2+^ for the first time. Simulations on wild type DHBPS and mutant complexes have depicted a possible path traveled by ions into the active site, besides the support provided by conserved residues for the positioning of metal ions.

## Results

### Molecular dynamics of DHBPS-Ru5P complex

**Figure 1:**
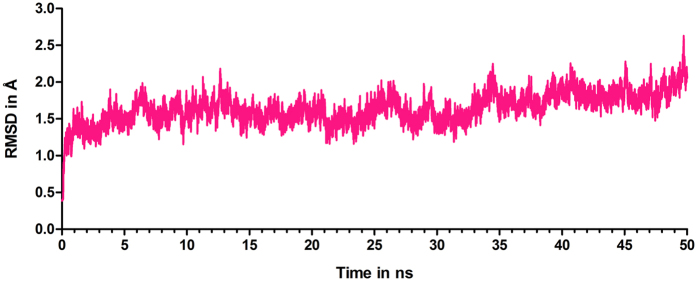
RMSD of the backbone atoms of DHBPS-Ru5P complex in the MD simulation, in comparison to the crystal structure.

Multiple MD simulations (in triplicate) are carried out on a system, where the crystal structure of DHBPS-Ru5P complex is present along with explicit water molecules and ions (Mg^2+^, Na^+^, and Cl^−^), by changing the initial velocities. In all of these simulations, Mg^2+^ has entered the active site. In two simulations, it has entered the active site of one monomer while, in the third simulation, it has entered the active sites of both monomers. Results of the simulations, in which Mg^2+^ has entered the active site of a monomer, are described here as these encompass the changes associated with the presence and the absence of ions in the active sites. During simulation, we have monitored parameters like volume, pressure, temperature, density, and energy to ensure the stability of the system. We have compared the conformations of DHBPS sampled during 50 ns of MD simulation by superimposing these on the crystal structure. Root mean square deviation (RMSD) of the conformations sampled during production simulation has been found to be fluctuating around an average of 1.7 Å within a narrow range (Fig. 1). Radius of gyration values are also stabilized suggesting that the dimer complex has preserved its folded form over the course of 50 ns (Supplementary Fig. S3). To understand the dynamics of C_α_ atoms of the protein, root mean square fluctuations (RMSF) are calculated, and are presented in Supplementary Fig. S4. The results indicate that the structure of DHBPS-Ru5P complex is stable, and can be used for further analysis. The plots of RMSD and radius of gyration versus time, for other two simulations are provided in Supplementary Fig. S5.

Additional MD simulations of DHBPS-Ru5P complex with neutral His-154 show that an ionic interaction between the phosphate group of Ru5P and the guanidine group of Arg-38 is lost after 15 ns of simulation in both the monomers (Supplementary Fig. S6). Loss of this interaction increases the distance between center of mass of loop-1 residues and Ru5P atoms from 8.5 Å to 13 Å (Supplementary Fig. S7), leading to a partial opening of the loop-1. Further, Mg^2+^ could not enter the active site of any of the monomers of DHBPS-Ru5P complex, suggesting the existence of His-154 in the protonated form.

#### Interactions of DHBPS with Mg^2+^

**Figure 2:**
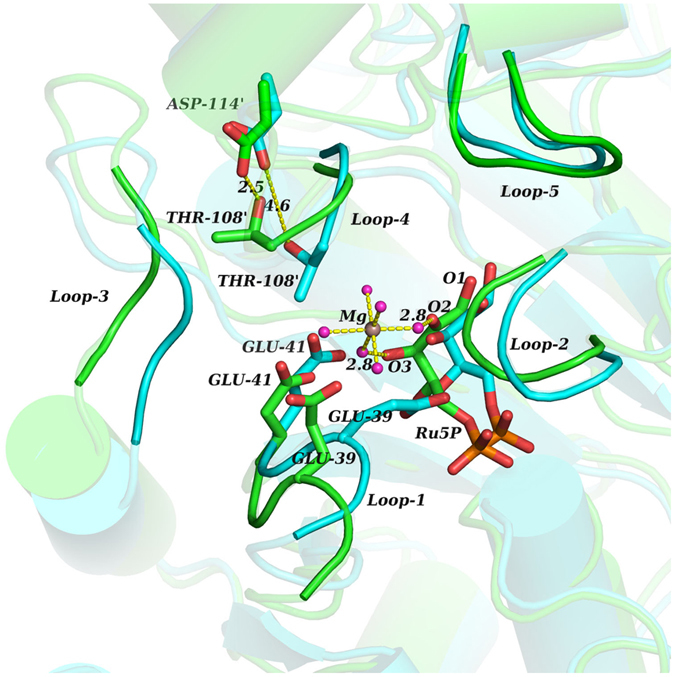
Conformational changes in the loops associated with the entry of Mg^2+^ into the active site of monomer-A. Superimposition of the crystal structure (cyan) and the last structure of 50 ns MD simulation (green) of DHBPS-Ru5P complex.

In a system of DHBPS-Ru5P complex prepared for MD simulations, every Mg^2+^ has six water molecules in its first water shell forming co-ordinate bonds. At the beginning of MD simulations, all of the Mg^2+^ are more than 10 Å away from the protein. However, at the end of 50 ns simulation one Mg^2+^ has entered the active site of monomer-A, and has stayed at a distance of 5–6 Å from O2 and O3 of Ru5P. This Mg^2+^ has been in an octahedral coordination shell with surrounding water molecules during the entire period of simulation (Supplementary Fig. S8). To understand the conformational changes associated with the movement of Mg^2+^ in the active site, we have superimposed the last structure of 50 ns simulations with the crystal structure (Fig. 2).

**Figure 3:**
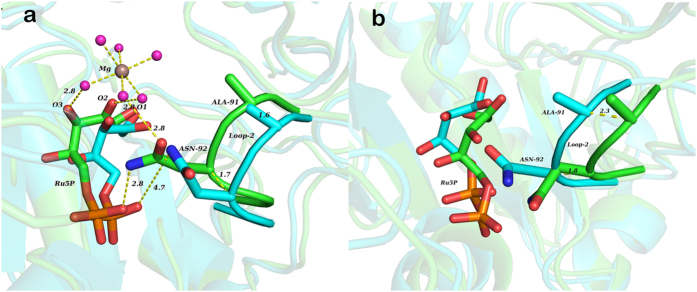
Position of the loop-2 in the active site of monomer-A and monomer-B during the MD simulation of 50 ns. (**a**) Superimposition of first (cyan) and 50 ns structure (green), showing monomer-A active site. (**b**) Superimposition of first (cyan) and 42 ns structure (green), showing monomer-B active site. Distances are shown in Å.

Analysis of the active site of monomer-A reveals that at 50 ns, C_α_ atoms of loop-1 residues are deviated sideways by a distance of 1.5 Å to 3.2 Å from that of the crystal structure. C_α_ atoms of Loop-3 residues are also moved sideways by a distance of 1.5 Å to 3.6 Å. These loop movements are away from Ru5P. In line with above loops, the loop-4 of monomer-B also moves away from Ru5P. In contrast to loop-1 and loop-3, loop-4 does not retain its original conformation as it varies in the side chain as well as backbone conformation. Thus, all the three loops - loop-1, loop-3, and loop-4 that are protruded inside the active site of crystal structure, are retracted backwards in the 50^th^ ns structure of simulations. Unlike these loops, loop-2 has shown movement into the active site. In monomer-A, C_α_ atoms of Ala-91 and Asn-92 of this loop have shifted into the active site by a distance of 1.6 Å and 1.7 Å, respectively from their initial position (Fig. 3a). Asn-92 of this loop interacts, through its sidechain oxygen, with Mg^2+^ entering the active site, as well as through its sidechain nitrogen, with one of the oxygens of a phosphate group of Ru5P. Once formed, these interactions are continued throughout the simulation period (Supplementary Fig. S9). In addition to these interactions, sidechain oxygen of Ser-90 and backbone nitrogen of Ala-91 form transient polar interactions with the water molecules that are surrounding the Mg^2+^. However, in monomer-B, loop-2 is shifted away from the active site. C_α_ atoms of Ala-91 and Asn-92 have moved away from the active site by a distance of 2.3 Å and 1.8 Å, respectively from their original position (Fig. 3b). The different behavior of loop-2 in monomer-A and monomer-B may be attributed to the presence of Mg^2+^ in the vicinity. Unlike in monomer-A, Asn-92 in monomer-B is unable to form hydrogen bond with the phosphate oxygen for the longer period, as observed in multiple simulations.

**Figure 4:**
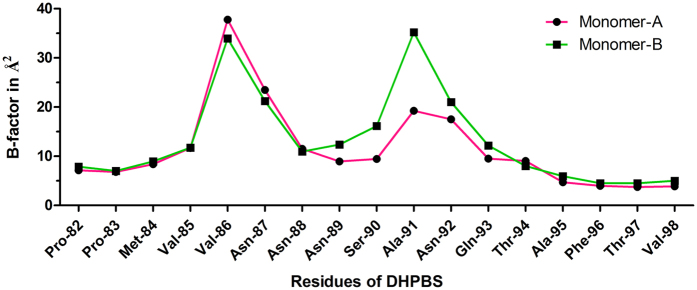
Flexibility of the loop-2 residues during the MD simulation. The figure shows B-factors of the loop-2 residues in monomer-A (pink) and monomer-B (green) of DHBPS-Ru5P complex.

We have analyzed backbone flexibility of loop-2 residues during 50 ns of simulation by calculating B-factors. It has been observed that residues 89–93 are less flexible in monomer-A compared to that in monomer-B (Fig. 4). Substrate conformations are also observed to be compact in monomer-A while these are variable in monomer-B, as observed in multiple MD simulations. In the active site of monomer-A, O3 of Ru5P shows hydrogen bond interaction with the side chain of Asp-43. This interaction has started after the entry of ions into the active site, and continued throughout the simulation. However, it is not observed in monomer-B (Supplementary Fig. S10).

#### Entry of Mg^2+^ into active site: Loop-4 as lid

**Figure 5:**
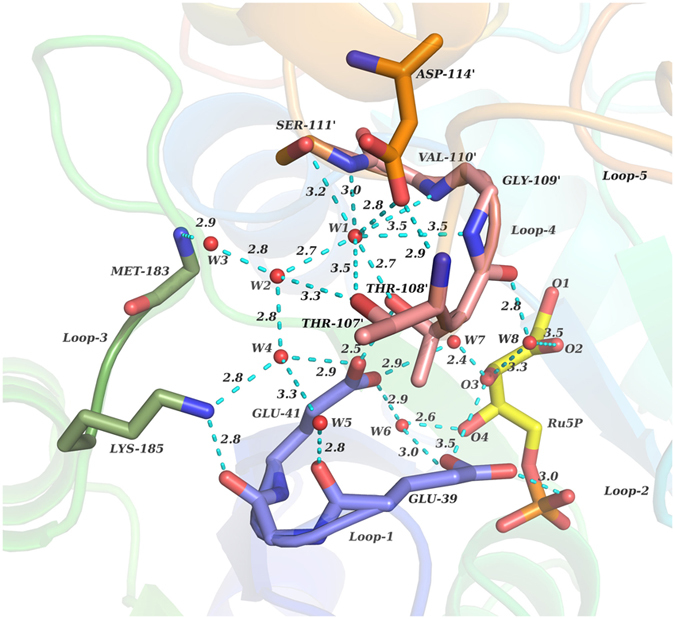
Interactions in the active site of DHBPS-Ru5P crystal structure complex. The figure shows hydrogen bonds formed within the residues of loop-1, loop-3, loop-4, water molecules and Ru5P. Water molecules, W1, W2, W3, W4, W5, W6, W7 and W8 are shown in red spheres. Distances are shown in Å.

During simulations, conformational changes in loop-1 and loop-4 are associated with the entry of Mg^2+^ into the active site of monomer-A. In the initial phase of simulation, the water molecule, W6, becomes unstable, and moves away from its position (Fig. 5). It leads to loss of interactions of Glu-39 and Glu-41 with Ru5P, as these are formed via the W6. Due to the absence of strong interactions with other residues and Ru5P, Glu-39 moves away from Ru5P and becomes exposed to the solvent. Glu-41 also loses its interaction with Thr-108′ of loop-4. This event is then followed by the movement of Mg^2+^ towards monomer-A. Positively charged Mg^2+^ is attracted by the negatively charged residues of loop-1. Asp-37 being exposed to the solvent, initiates electrostatic interactions with the incoming Mg^2+^. Transient and subsequent interactions with Asp-37, Glu-39, and Glu-41 guides Mg^2+^ towards the active site. Before entering the active site, Mg^2+^ is stabilized by both the exposed residues, Glu-39 and Glu-41.

**Figure 6:**
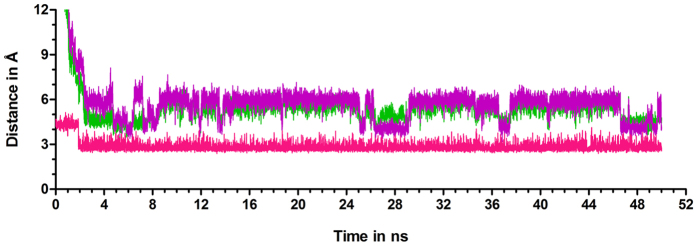
Interactions associated with the entry of Mg^2+^ into the active site of DHBPS. Distance between Mg^2+^ and O2/O3 of Ru5P are represented by purple and green, respectively. Mg^2+^ moves into the active site after the formation of hydrogen bond between sidechain oxygen atoms of Thr-108′ and Asp-114′ (shown in pink).

**Figure 7:**
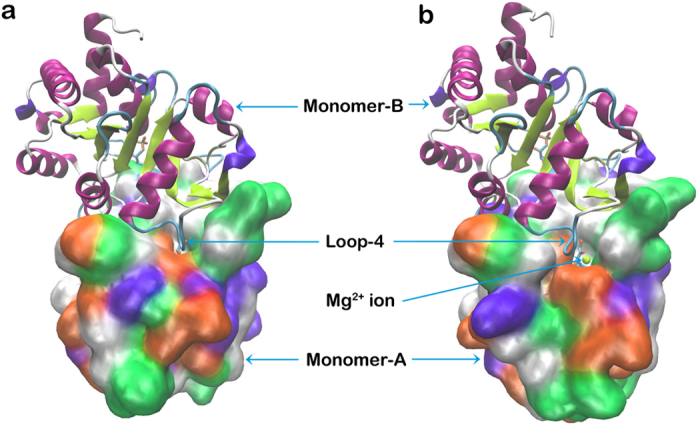
Conformational change in Loop-4 as Mg^2+^ enters active site. First **(a)** and last **(b)** MD simulation structure of DHBPS-Ru5P complex. Monomer-A is shown in solid surface having residue type coloring scheme while Monomer-B is shown in cartoon representation. Magnesium ion is shown as a sphere in yellow. Entry of Mg^2+^ into the active site is blocked by the loop-4 of monomer-B.

Entry of this ion into the active site of monomer-A is blocked by loop-4 of monomer-B, especially by Thr-107′ and Thr-108′. It has been noted that O2 and O3 of Ru5P act as points of contact for Mg^2+^ to enter the active site. However, movement of loop-4 residues is essential to make O2 and O3 atoms of Ru5P accessible to Mg^2+^. This is achieved by the formation of hydrogen bond between the side chain oxygen of Thr-108′ and the side chain oxygen of Asp-114′ (Fig. 2). The hydrogen bond between side chains of Thr-108′ and Asp-114′ occurs at the expense of conserved water molecule, W1 (Fig. 5). In the first structure of simulation, both the residues are in contact with each other by forming water (W1) mediated hydrogen bond. During MD simulations, the water molecule, W1, becomes unstable, and pulls the interacting hydroxyl group of Thr-108′ to take its position, and subsequently leaves its place. The hydroxyl group of Thr-108′ then initiates hydrogen bond interactions with backbone nitrogen atoms of Gly-109′, Val-110′, and Ser-111′ and side chain oxygen of Asp-114′ (Supplementary Fig. S11) leading to reorientation and opening of loop-4, thereby creating space for the entry of Mg^2+^. Mg^2+^ then moves by 3 Å towards the Ru5P from a distance of 8–9 Å, and gets stabilized at 5–6 Å by interacting with the O2 and O3 atoms of Ru5P (Fig. 6). However, above mentioned interactions are not observed in the crystal structure of DHBPS-Ru5P complex, and point of entry of metal ion is covered by residues of loop-4 (Fig. 7). Thus, it appears that this loop-4 of one monomer acts as “lid” over the active site of another monomer.

### Molecular dynamics of mutant DHBPS-Ru5P complexes

MD simulations of DHBPS-Ru5P complex suggest that movement of Mg^2+^ in the active site has been channeled by the formation of hydrogen bond between Thr-108 and Asp-114. To substantiate the observed set of events, we have mutated these residues in both monomers of DHBPS-Ru5P complex, and have observed the movement of Mg^2+^ during MD simulations. RMSD analysis of these mutant DHBPS-Ru5P complexes shows that these complexes are stable during MD simulations (Supplementary Fig. S12).

#### Thr108Ser mutant DHBPS-Ru5P complex

At the end of 50 ns MD simulation of Thr108Ser mutant DHBPS-Ru5P complex, one Mg^2+^ is stabilized in the active site of monomer-A. Ser-108′ forms hydrogen bond with the carboxylic group of Asp-114′ through the sidechain hydroxyl group during the simulation. This hydrogen bond is formed in the initial few picoseconds, and continued till 50 ns (Supplementary Fig. S13), resulting in the formation of a necessary cavity for Mg^2+^ to enter the active site. After entering the active site, Mg^2+^ is stabilized at its position throughout 50 ns simulation. However, in monomer B, one of the Mg^2+^ is attracted towards the acidic active site loop, and is stabilized at a distance of ~ 10 Å from Ru5P without entering the active site. The hydrogen bond between Ser-108′ and Asp-114′ is not observed, keeping loop-4 at its position, and forbidding the entry of ion (Supplementary Fig. S14). This simulation supports that formation of hydrogen bond between Thr-108 and Asp-114 is essential for the entry of ions into the active sites of DHBPS.

#### Thr108Val mutant DHBPS-Ru5P complex

In this study, we have mutated Thr-108 to valine. Valine has the same number of heteroatoms in the sidechain as that of threonine, but it lacks the side chain hydroxyl group. It is expected that Mg^2+^ may not enter the active site as the absence of hydrogen bond between Val-108 and Asp-114 shall not reorient loop-4. MD simulations depict that one Mg^2+^ has entered the active site of monomer-A, and is stabilized, contrary to our expectations. The backbone nitrogens of Val-108′ and Thr-107′ form strong hydrogen bonds with the side chain of Asp-114′. Before the entry of Mg^2+^ into the active site, the hydroxyl group of Thr-107′ also interacts with the carboxyl group of Asp-114′. These interactions have caused flipping of dimethyl group of valine as well as reorientation of loop-4, opening a space for metal ions to enter. Mg^2+^ enters the active site, and is stabilized by the formation of interactions with O3 and O4 of Ru5P, instead of O2 and O3 of Ru5P as observed in the crystal structure. Further, the conformation of Ru5P in this mutant complex is unlike that of the crystal structure. These observations suggest that mutation of Thr-108 to valine allows entry of Mg^2+^ into the active site, but it may not result in the proper orientation of Mg^2+^ and Ru5P, as required for the catalysis.

#### Asp114Ser mutant DHBPS-Ru5P complex

In another mutational study, we have replaced Asp-114 with serine. The side chain of serine is shorter in length by one carbon atom, and it may not form interactions like aspartic acid as observed in the wild type. During MD simulations of this complex, Mg^2+^ does not enter the active site of any of the monomers. In monomer-A, one of the ions gets stabilized at 11 Å from the substrate without entering active site throughout simulations as the hydrogen bond between Thr-108′ and Ser-114′ is not formed (Supplementary Fig. S15).

#### Asp114Leu mutant DHBPS-Ru5P complex

In this mutational study, Asp-114 in DHBPS-Ru5P complex has been mutated to leucine. MD simulations show that none of the Mg^2+^ interacting with the acidic active site loop has moved into the active site of both the monomers. As Leu-114′ lacks sidechain carboxylic group, it cannot form the hydrogen bond with the loop-4 residues viz. Thr-107′, Thr-108′, and Gly-109′. It supports that these interactions are essential for the opening of loop-4, suggesting loop-4 as a “lid” over the active site.

#### Glu39Ala, Glu41Ala, and His154Ala mutant DHBPS-Ru5P complexes

The root mean square deviations of these mutant complexes have been shown to be stable during MD simulations (Supplementary Fig. S16). The analysis of results shows that a Mg^2+^ enters the active site in a few of the MD simulations. However, the conformations of Ru5P, and the positions of stabilized Mg^2+^ does not superpose with those of wild type (Supplementary Fig. S17). Further, the formation of hydrogen bond between Thr-108 and Asp-114 has been observed before the entry of Mg^2+^.

## Discussion

DHBPS is vital to the survival of many pathogens and thus considered as a potential drug target to design antibacterial agents. Mechanistic and dynamic details of the binding of substrate, ions, and the role played by residues are required to understand the functioning of DHBPS, as well as for the effective design of novel inhibiting agents. In a first of its kind, we have performed conformational analysis of DHBPS and Ru5P in the presence of Mg^2+^ ions using MD simulations. During 50 ns of simulation of DHBPS-Ru5P complex, loop-2 in the active site of monomer-A has been found to be ordered and stable. It forms interactions with entered Mg^2+^ and phosphate oxygen atoms of Ru5P. However, loop-2 in the active site of monomer-B is ordered but less stable, and does not form interactions with the substrate for most of the time of simulation. Similar behavior of the loop-2 is reported in Ru5P complexed crystal structures. In DHBPS-Ru5P complex of *V. cholera*^26^ and *C. albicans*^20^, this loop is partially disordered whereas, in DHBPS-Ru5P-Zn^2+^ complex of *V. cholera*^26^ and *M. jannaschii*^14^, it is in ordered conformation, forming weak water mediated interactions with the phosphate group of Ru5P. However, in MD simulations this loop forms strong and direct interactions with the phosphate group via Asn-92. This study highlights the role of loop-2 in positioning and stabilization of Mg^2+^ at M2 position along with the stabilization of phosphate group of Ru5P. In the absence of ions, this loop does not significantly contribute to the stabilization of substrate as its residues remain flexible and interact transiently.

MD simulations show substrate, Ru5P to be flexible in monomer-B but adopts a compact conformation in monomer-A. Interaction analysis of Ru5P reveals that Ru5P undergoes Mg^2+^ induced conformational change such that its O2 and O3 hydroxyl groups reorient to interact with the entered Mg^2+^. This reorientation is stabilized by the formation of hydrogen bond between O3 of Ru5P and sidechain oxygen of Asp-43. These events are supported by the observation that O3 of Ru5P moves by ~1 Å towards the sidechain oxygen of Asp-43 in the presence of ions upon comparison of DHBPS-Ru5P and DHBPS-Ru5P-Zn^2+^ complexes. These two oxygen atoms then become a part of strongly bonded coordinate geometry of Mg^2+^ providing conformational rigidity to Ru5P in monomer-A. The high flexibility of Ru5P in the monomer-B can be attributed to the absence of ion in the active site, as well as weaker binding of its hydroxyl groups with surrounding residues such as Asp-43. In the absence of Ru5P (i.e. O2 and O3 atoms), either phosphate or sulphate is present along with water molecules for necessary interactions with incoming Mg^2+^ 14,17,18,25,26. Even in the apo structure of *V. cholera,* water molecules present in the active site superpose well with hydroxyl oxygen atoms of Ru5P^26^.

In this study, we have performed MD simulations of DHBPS-Ru5P complexes using protonated His-154, and have found that His-154 forms stable interactions with a phosphate group of Ru5P, similar to that in the crystal structure. In another simulation of DHBPS-Ru5P complex, having neutral His-154, these interactions are lost resulting in destabilization of Ru5P. Interactions of metal ion at position M1 with residues like His-154 are found to be conserved in separate MD simulations of metal bound DHBPS-Ru5P complex having neutral His-154. However, these interactions are lost in the presence protonated His-154, destabilizing ion at M1 as well as Ru5P. In addition, pKa prediction results show that His154 is protonated in DHBPS-Ru5P complex while it is neutral in DHBPS-Ru5P-Zn^2+^ complex. Our study finds that only one Mg^2+^ gets recruited in each of the active sites of DHBPS monomers, and it occupies the position close to M2 of DHBPS-Ru5P-Zn^2+^ complex. The second Mg^2+^ that occupies the M1 position, coordinating with His-154, does not enter the active sites as protonation states of His-154 are not changed on the fly during the MD simulation. It is likely that first ion that enters the active site binds transiently at M2 position before moving to M1. This transfer, however, may require changes in the protonation state of His-154 from protonated to a neutral state, leading to the stabilization of ion at the position M1. Subsequently entering ion is then positioned at M2 site. Entry and stabilization of Mg^2+^ at a position close to M2 can be attributed to the same protonation state of O2 and O3 atoms of Ru5P in DHBPS-Ru5P and DHBPS-Ru5P-Zn^2+^ complexes. This is because of the fact that pKa of the hydroxyl groups of Ru5P is much higher (~12), suggesting that these groups are less likely to be deprotonated even if coordination to magnesium decreases their pKa values.

**Figure 8:**
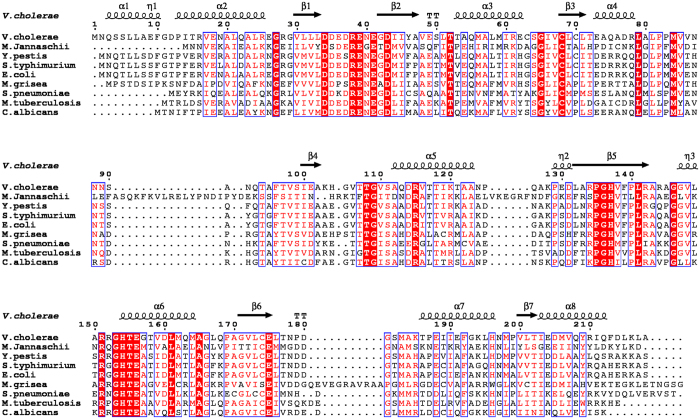
Sequence alignment of DHBP synthases from different species. Residues strictly conserved are highlighted in red while almost conserved ones are typed in red. The secondary structure of the *V. cholerae* is shown above the sequences.

Formation of the hydrogen bond between the side chains of Thr-108 and Asp-114 is related to the transfer of ion into the active site, as observed during MD simulations. This interaction subsequently creates other hydrogen bond interactions between Thr-108 and loop-4 residues viz. Gly-109, Val-110, and Ser-111 providing stability to the flipped Thr-108. These residues are also involved in the reorientation of loop-4 along with the transfer of ion. This loop-4 acts as a “lid” that opens up and allows Mg^2+^ to enter the active site of another monomer. Apo as well as Ru5P complexed structures of DHBPS, do not show the formation of this hydrogen bond, thus closing the entry point for metal ions. Thus, the “lid” is closed in DHBPS-Ru5P-Zn^2+^ complexes, trapping ions in the closed state. We have analyzed the conserved nature of residues involved in ion transfer *viz.* Asp-114, Thr-108, Gly-109, Val-110, and Ser-111 across DHBPS of other microorganisms by sequence alignment (Fig. 8). The sequence identity of DHBPS between these microbial species and *Vibrio cholerae* ranges from 30% to 67%. Asp-114 is found to be mostly conserved. Only in *S. pneumoniae,* Asp-114 is replaced with another amino acid, i.e. glutamic acid (Fig. 8), which, however, conserves the side chain carboxylic oxygen atoms required for necessary interactions with Thr-108. MD simulations of Asp114Ser mutant complex show that ions do not enter the active site of both monomers, and may result in a reduction of catalytic activity of DHBPS, as seen in the same mutant of *M. jannaschii*^27^. It may be stated that DHBPS of *Vibrio cholarae* shows 30% amino acid identity with that of *M. jannaschii*, and both enzymes require Mg^2+^ as a cofactor, and follow the same mechanism of catalysis^14^,^26^. In another MD study, results of Asp114Leu mutation show that entry of ion into the active site is prohibited. These two mutational studies establish the role of Asp-114 in ion transfer, and thus explain its conservation among DHBP synthases. Thr-108, another residue, is strictly conserved among DHBP synthases (Fig. 8). In MD study of Thr108Ser mutant complex, results have shown that an ion has entered the active site of DHBPS, similar to that in wild type. However, same mutation has resulted in the loss of activity of DHBPS in *M. jannaschii*^27^. This contrary result may be explained by the fact that the sidechain methyl group of Thr-108 stabilizes the sidechain of Glu-39 by hydrophobic interactions^14^,^26^, which may not be achieved by serine mutant due to absence of methyl group. Sidechain carboxylic oxygen atoms of Glu-39 have been shown to interact with metal ions at positions M1 and M2^14^,^26^ and mutation of Glu-39 have resulted in the loss of activity of DHBPS in *M. jannaschii*^14^ as well as in *V. cholera*^26^. In another MD study of Thr108Val mutant DHBPS-Ru5P complex, Mg^2+^ enters the active site but it is stabilized away from M2 position, unlike Mg^2+^ in wild type complex, explaining the loss of activity and importance of conservation of Thr-108. However, the catalytic activity of Thr-108 and Asp-114 mutants in V. cholerae needs to be validated experimentally. Nevertheless, results of MD studies on the mutant complexes involving mutations viz. Glu39Ala, Glu41Ala, and His154Ala suggest varied conformations of Ru5P and positions of Mg^2+^, explaining the loss of the activity of DHBPS, as observed in experimental studies^26^. Other residues such as Gly-109, Val-110, and Ser-111 that are involved in hydrogen bonding with Thr-108 are also either strictly or mostly conserved (Fig. 8). Glycine (−109), is also proposed to play a structural role in *E. coli* DHBPS, and is predicted to have strained conformation for a non-glycine residue as there is no room for C_β_ atom^16^. Thus, it is expected that Gly-109 allows Thr-108 to change its conformation and form the hydrogen bond with Asp-114. It is likely that the replacement of Gly-109 by any other residue may forbid the formation of this bond due to steric hindrance. Similar amino acids at positions of Val-110 and Ser-111 may participate in a reorientation of loop-4 as MD studies have shown that these residues interact with Thr-108 through backbone nitrogen atoms. Thus, conservation of these residues and interactions substantiates mechanism of ion transfer and puts forward that it may exist in other microorganisms as well.

## Material and Methods

### Preparation of system for molecular dynamics

In this study, we have used DHBPS-Ru5P complex of *Vibrio cholerae*^26^. Structural coordinates of this complex have been obtained from protein data bank (PDB ID 4P77, resolution 2.04 Å). This structure is the homodimer of 431 residues (216 residues in chain A and 215 residues in chain B) with Ru5P bound in each of the active sites. The coordinates of residues- 90–91 of A-chain and residues 87–93 of B chain are missing in this structure. These residues have been modeled from the *Vibrio cholera* DHBPS-Ru5P-Zn^2+^ structure by direct transfer of coordinates (PDB ID 4P8E, resolution 2.04 Å)^26^. The complex has been prepared for MD simulations using various modules of AMBER14^28^,^29^. Initially, the protonation states of amino acids are determined in the presence of co-crystallized water molecules at pH 7.0 using PROPKA module and then verified manually^30^,^31^. Four of the residues, Glu-41, Asp-43, His-137, and His-154 are predicted to be protonated. Among these, visual inspection reveals that Glu-41 is exposed to the solvent, and Asp-43 forms anion-pi interactions (3.5 Å) with positively charged His-154, depicting these to be deprotonated. Then, according to the protonation states, hydrogen atoms have been added to all residues using LEaP module of AMBER14.

In Antechamber, for Ru5P, the AM1-BCC method has been utilized to calculate partial atomic charges^32^,^33^, and the general amber force field (GAFF) is used to assign the parameters^34^. Protein topology has been prepared with ff14SB force field^35^. Protein complex with its crystal waters is then solvated with TIP3P water model^36^ in a truncated octahedral box of a volume of 579921.85 Å^3^, and the edge of the box is 8.5 Å from the protein. The system is neutralized by adding a requisite number of Mg^2+^ and Na^+^ ions. Subsequently, Mg^2+^ and Cl^−^ ions have been added to make 0.1 M concentration of the system.

Ions have been added in such as way that they are no closer than 10 Å from the complex, and are apart by 5 Å from each other. It avoids artifacts whereby ions may enter the active site because of their placement nearby the complex. A compromise (CM) set of ion parameters that reproduce the experimental and relative hydration free energy (HFE), and coordination number (CN) values have been used to define the divalent ions^37^. Force field parameters, fitting solvation free energies, radial distribution functions, ion-water interaction energies, and crystal lattice energies and lattice constants for non-polarizable spherical ions, developed by Joung and Cheatham are used for the monovalent ions^38^,^39^. A topology file containing force field parameters, and a co-ordinate file containing structural information of the complex, are then used as input to perform the MD simulations using pmemd.cuda module of AMBER14^29^.

At first, a two-stage energy minimization process has been used to allow the system to reach an energetically favorable conformation. In the first stage, positions of protein, Ru5P, and ions are restrained with a harmonic force constant of 10 kcal/mol/Å^2^ while, explicitly added water molecules are allowed to move, and adjust their orientations. In the second stage, the force constant is reduced to zero, allowing the system to accommodate protein, Ru5P, water, and ions. The temperature of the system is then increased to 300 K using NVT ensemble over the period of 100 ps. In this step protein, Ru5P, and ions are restrained by a force constant of 0.5 kcal/mol/Å^2^. Subsequently, the system is equilibrated such that it has a uniform density around 1 g/cm^3^, and has achieved stable RMSD of protein during a period of 10 ns. The sampling MD simulations are then performed in an isothermal-isobaric ensemble (NPT, P = 1 atm, and T = 300 K) for 40 ns. The time step is kept at 2 fs, while the trajectory is recorded every 4 ps. Simulation is repeated three times by changing the initial velocity. Langevin thermostat and Berendsen barostat are used for temperature and pressure coupling, respectively^40^,^41^. SHAKE algorithm is applied to constrain all the bonds containing hydrogen atoms^42^. A non-bonded cutoff is kept at 8 Å, and long range van der Waals interactions have been treated by Particle Mesh Ewald (PME) method^43^. Discovery Studio and Pymol have been used to prepare files, visualize 3D structures, and create graphics^44^,^45^ while, VMD and Ptraj program are used to visualize trajectories, compare residues and ion movements, and compute interactions^28^,^46^.

### Mutation of residues

During the simulations, amino acids Thr-108 and Asp-114 are observed to be critical for the movement of the metal ions in the active site. In DHBPS-Ru5P complex, these residues are mutated to serine (Thr108Ser and Asp114Ser) or valine (Thr108Val) or leucine (Asp114Leu) in LEaP module of AMBER14. As both residues do not form direct interaction with Ru5P, it is believed that their mutations do not affect the binding of substrate in the active site of DHBPS. In addition, catalytic residues viz. Glu39, Glu41, and His154 of DHBPS, have been mutated to alanine to understand the structural changes leading to loss of catalytic activity of the DHBPS. For MD simulations, the backbone of the mutated residues has been kept unchanged while side-chains are built into the structures with default geometry. The same protocol is followed to prepare systems for each mutation, and then subjected to MD simulations for 50 ns, in duplicate.

### Sequence Alignment

Multiple sequence alignment is performed by aligning *V. cholerae* DHBPS with sequences from other species using default values in Clustal Omega^47^. DHBPS-Ru5P complex (PDB ID 4P77) is used to display the secondary structure on the top of the alignment. The alignment figure has been generated through ESPript 3 web server^48^, using BLOSUM 62 score matrix^49^.

## Additional Information

**How to cite this article**: Shinde, R. N. *et al*. Molecular dynamics studies unravel role of conserved residues responsible for movement of ions into active site of DHBPS. *Sci. Rep.* **7**, 40452; doi: 10.1038/srep40452 (2017).

**Publisher's note:** Springer Nature remains neutral with regard to jurisdictional claims in published maps and institutional affiliations.

## Supplementary Material

Supplementary Information
