# Supplementary material for: Molecular dynamics studies unravel role of conserved residues responsible for movement of ions into active site of DHBPS
Source: Sci Rep. 2017 Jan 12;7:40452. doi: 10.1038/srep40452 (PMC5228156; doi:10.1038/srep40452)
Supplement: Supplementary Information [file srep40452-s1.doc]

**Supplementary Information**

**Molecular dynamics studies unravel role of conserved residues responsible for movement of ions into active site of DHBPS**

Ranajit Nivrutti Shinde,^1^ Subramanian Karthikeyan,^1,*^ and Balvinder Singh^1,*^

**
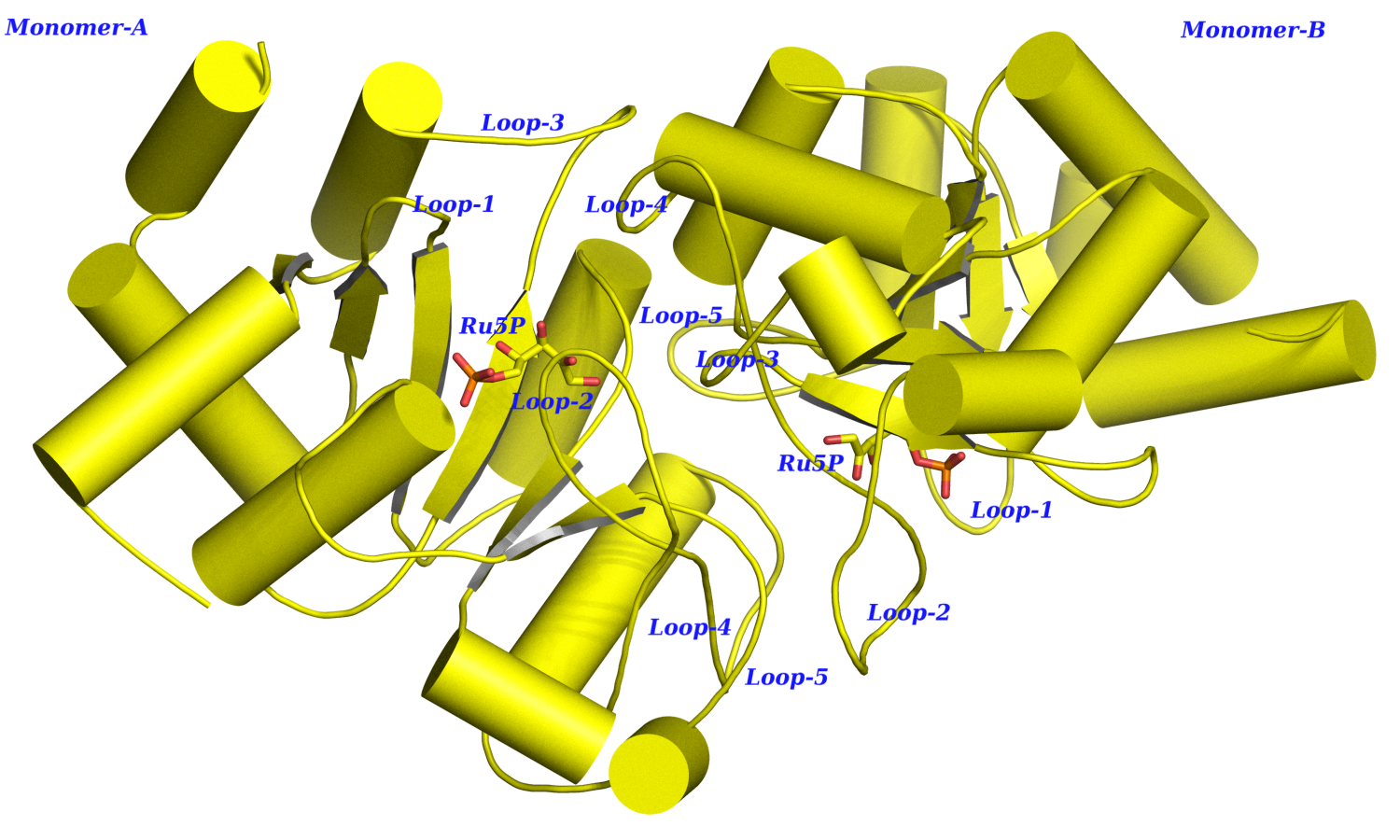
**

**Supplementary Figure S1.** **Active sites of DHBPS-Ru5P complex.** Loops adjacent to the active site of monomer-A and monomer-B are labeled. Substrate, Ru5P, in both active sites is shown in stick representation.


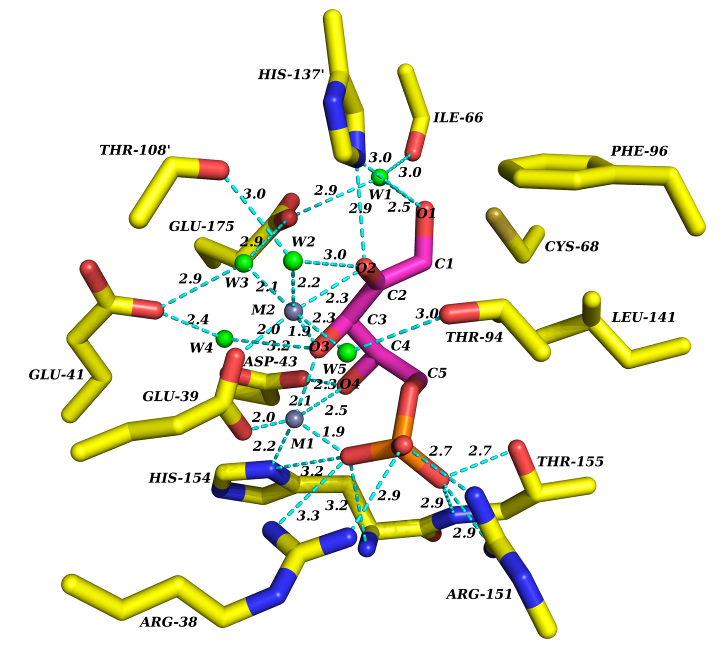


**Supplementary Figure S2**. **Active site of DHBPS-Ru5P-Zn^2+^ complex.** Dark gray spheres are Zn^2+^ ions at position M1 and M2. Substrate, Ru5P, is magenta, residues are yellow and water molecules are green. The dash line connects hydrogen bond forming atoms and distance between them is shown in Å. Residues of monomer-B are labeled with primes (Thr-108’ and His-137’).

**
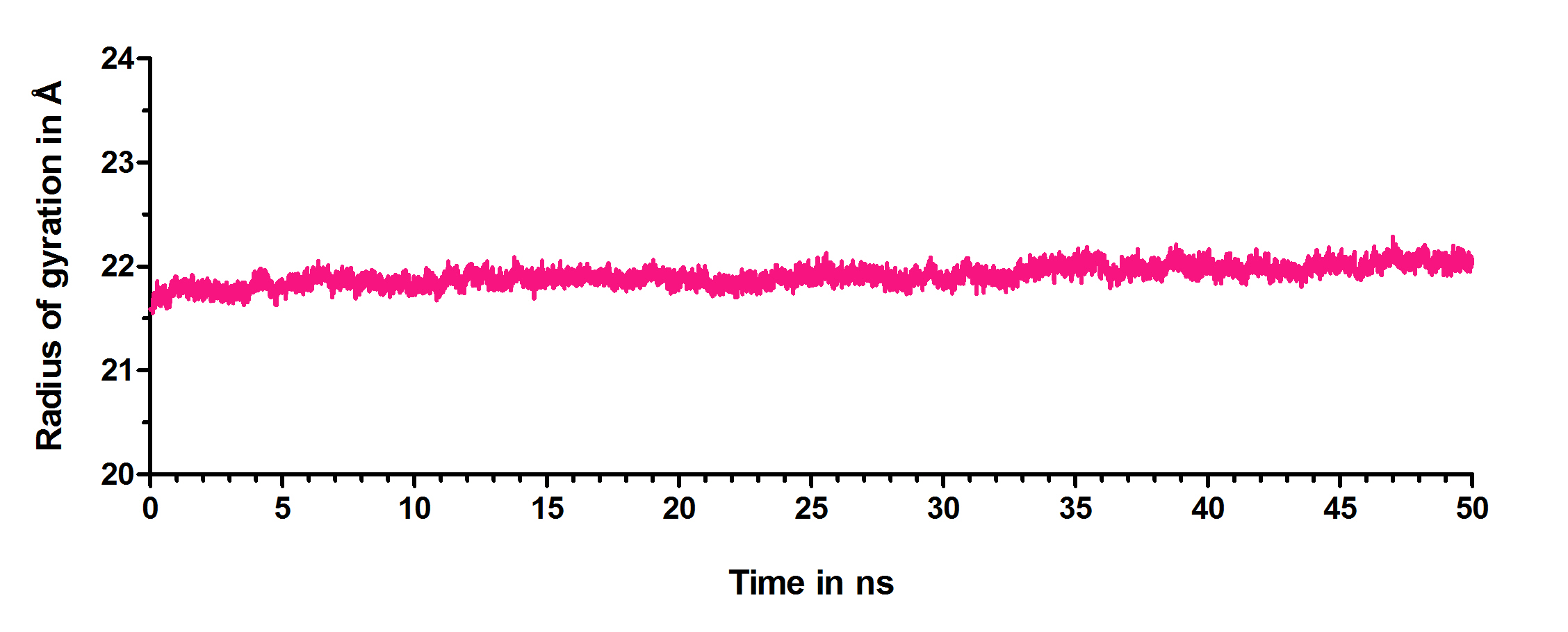
**

**Supplementary Figure S3.** Radius of gyration of C_α_ atoms of DHBPS-Ru5P complex in MD simulation of 50 ns.


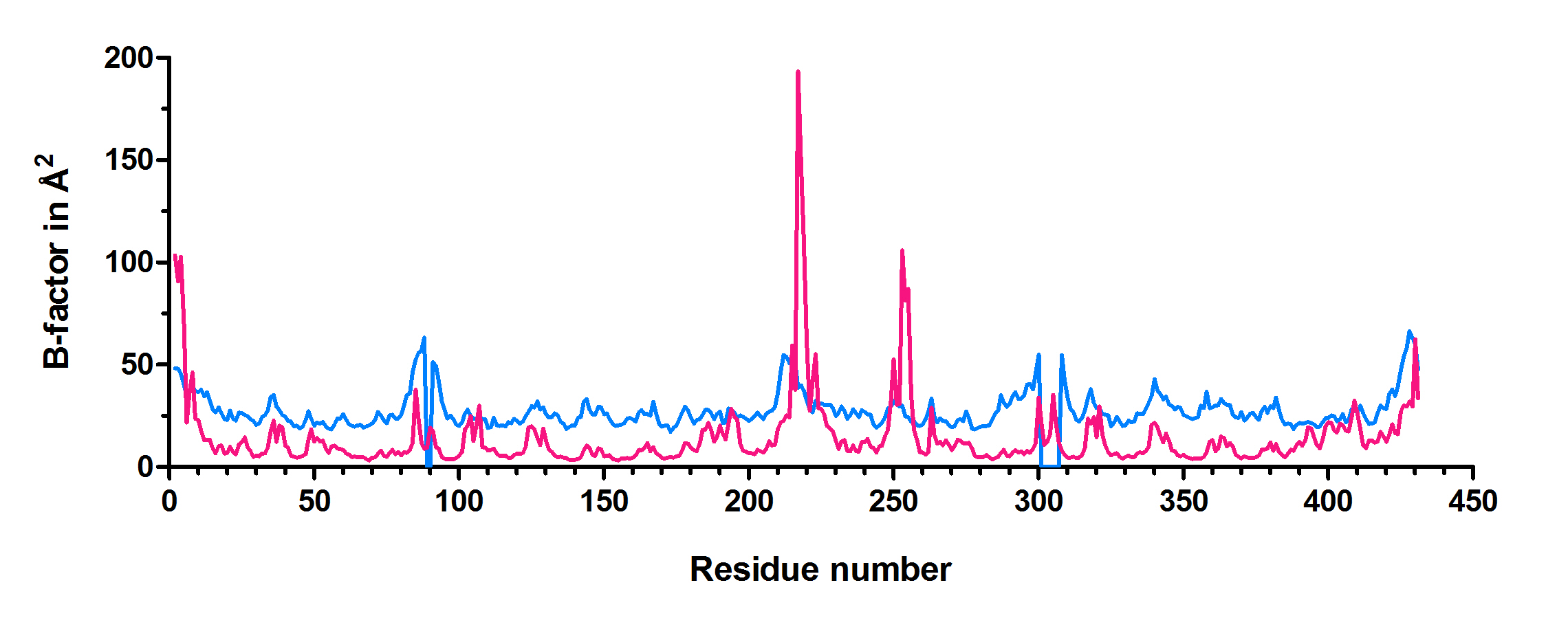


**Supplementary Figure S4.** **Flexibility of residues**. B-factor of C_α_ atoms of DHBPS residues in crystal structure (blue) and in MD simulation (pink). Residues 1-216 are of monomer-A whereas residues 217-431 are of monomer-B.


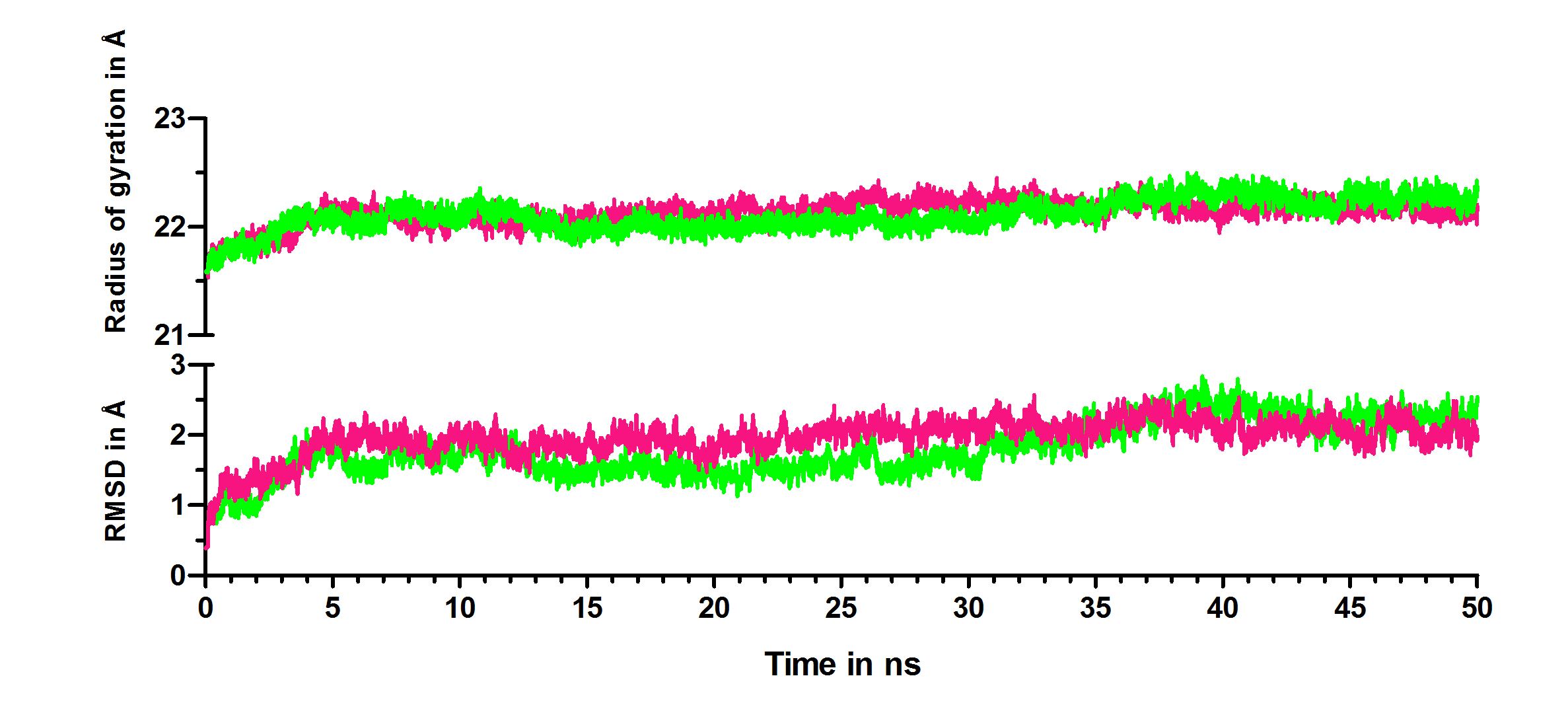


**Supplementary Figure S5.** Radius of gyration (C_α_ atoms) and RMSD (backbone atoms) of DHBPS-Ru5P complex in two separate MD simulations represented by pink and green colours.


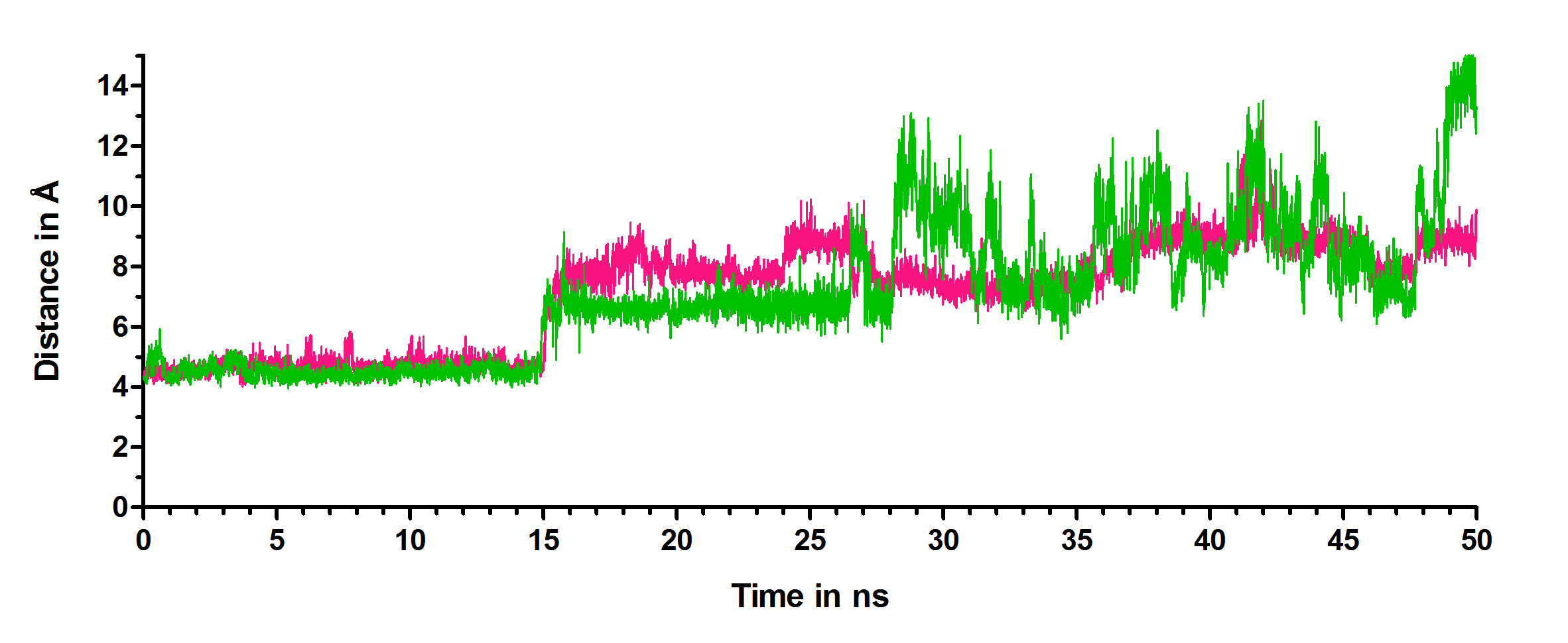


**Supplementary Figure S6: Loss of ionic interactions between Arg-38 and Ru5P during the MD simulation.** The figure shows distance between guanidino-carbon atom of Arg-38 and phosphorous atom of phosphate group of Ru5P in monomer-A (pink) and monomer-B (green) of DHPBS-Ru5P complex.


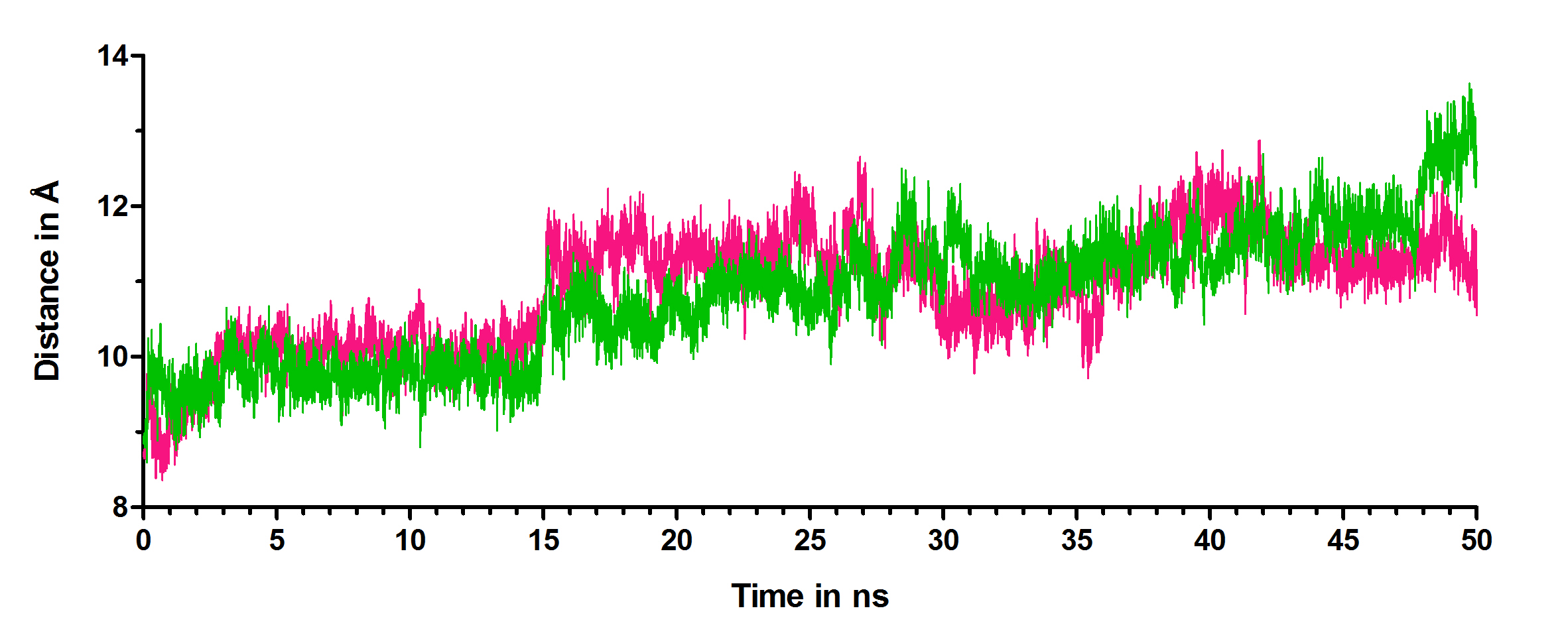


**Supplementary Figure S7: Movement of loop-1 away from Ru5P in the MD simulation.** The figure shows the distance between the center of mass of loop-1 residues (34-41) and Ru5P in monomer-A (pink) and monomer-B (green) of the DHPBS-Ru5P complex.


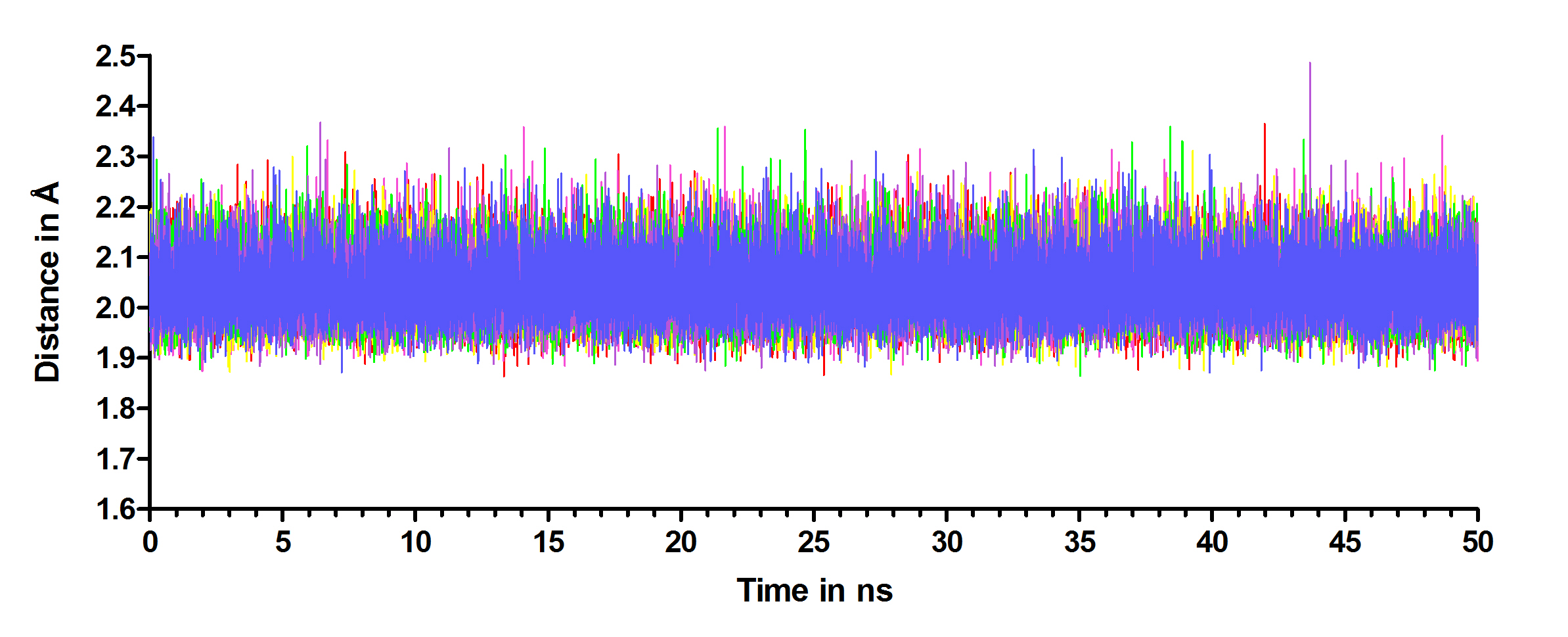


**Supplementary Figure S8.** **The octahedral coordination of the Mg^2+^ that enters the active site of DHBPS-Ru5P complex.** Figure shows distances between Mg^2+^ and the oxygen atom of six water molecules, represented by blue, green, purple, pink, yellow and red, that forms an octahedral coordination complex, throughout the simulation period of 50 ns.


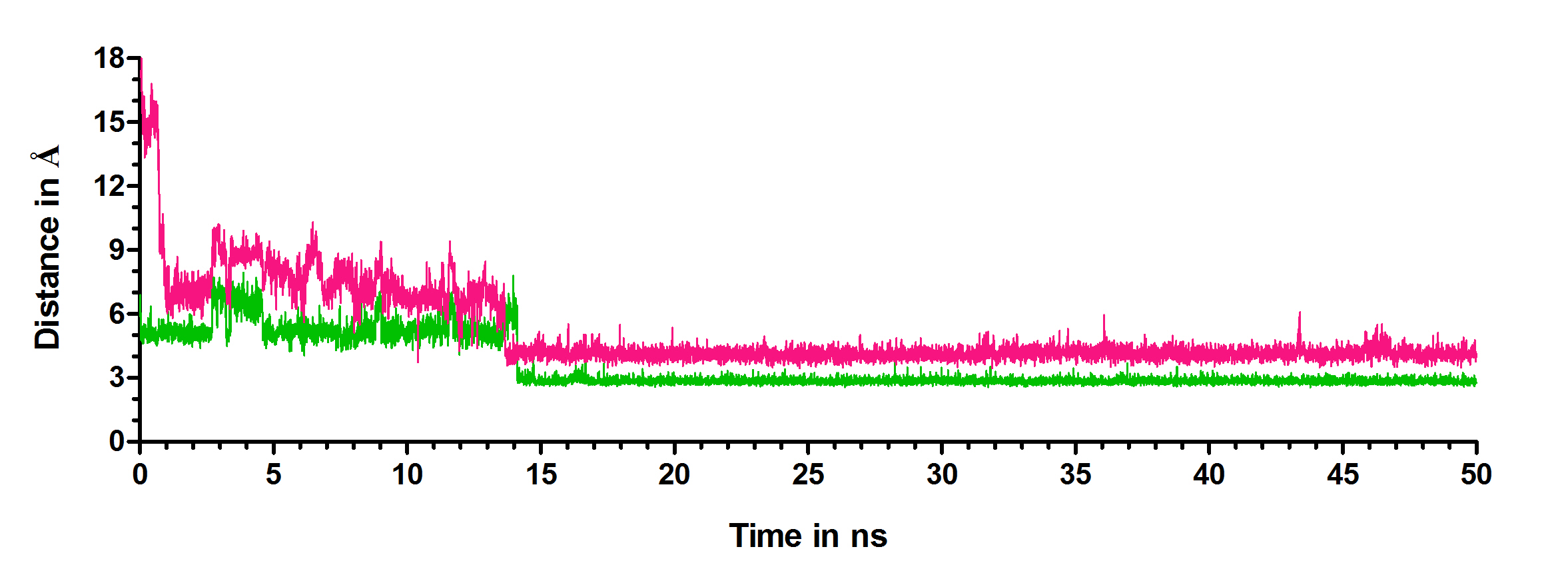


**Supplementary Figure S9.** **Interactions of loop-2 residue, Asn-92, during MD simulation**. Distance between the side chain oxygen of Asn-92 and Mg^2+^ is shown in pink. Distance between side chain nitrogen of Asn-92 and one of the oxygen atoms of a phosphate group of Ru5P is shown in green. Once formed Asn-92 continues its interactions with Mg^2+^ and phosphate oxygen of Ru5P throughout the simulation time in monomer-A.


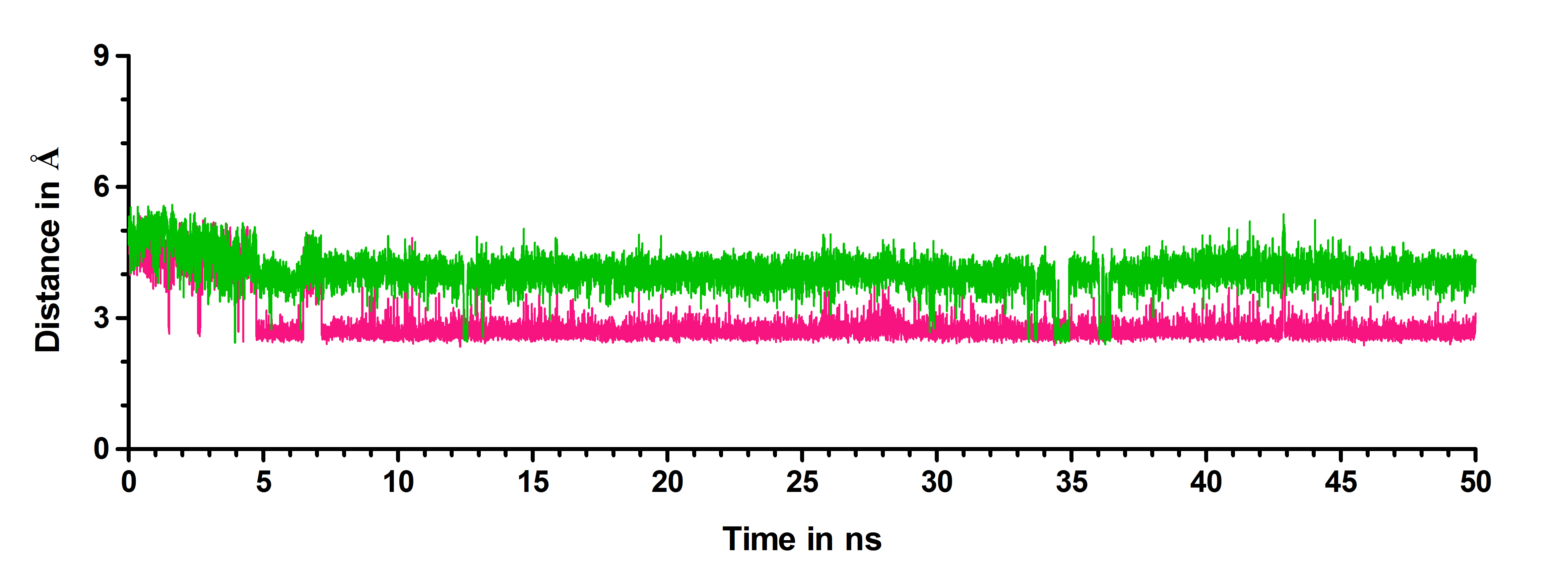


**Supplementary Figure S10.** **Interaction between Asp-43 and Ru5P during MD simulation**. Distance between side chain oxygen of Asp-43 and O3 oxygen of Ru5P in monomer-A (pink) and monomer-B (green).


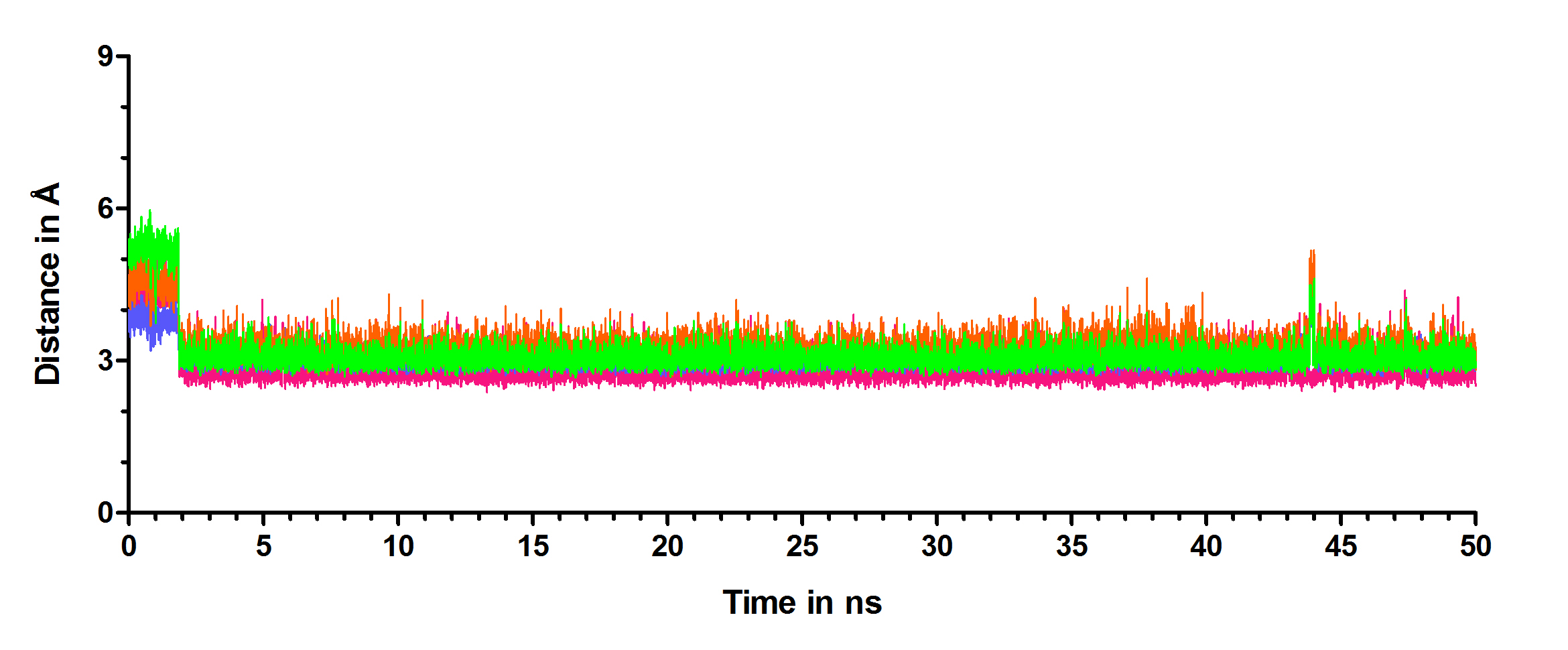


**Supplementary Figure S11.** Distance between hydrogen bond forming atoms of Thr-108’ and Gly-109’(blue)/Val-110’(orange)/Ser-111’(green)/Asp-114’(pink) in monomer-A of MD simulated DHBPS-Ru5P complex.


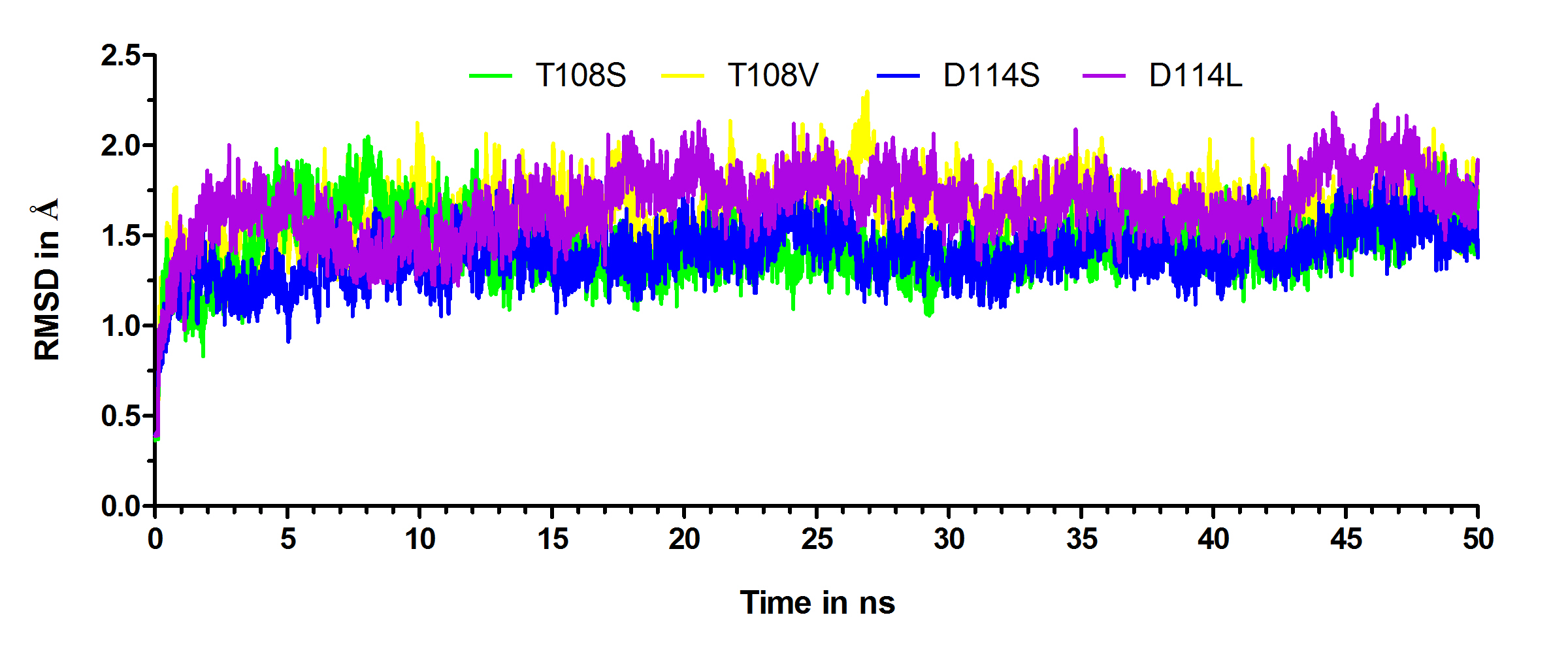


**Supplementary Figure S12. Stability of MD trajectories of mutant complexes.** Root mean square deviation of backbone atoms of mutant DHBPS-Ru5P complexes in MD simulation.


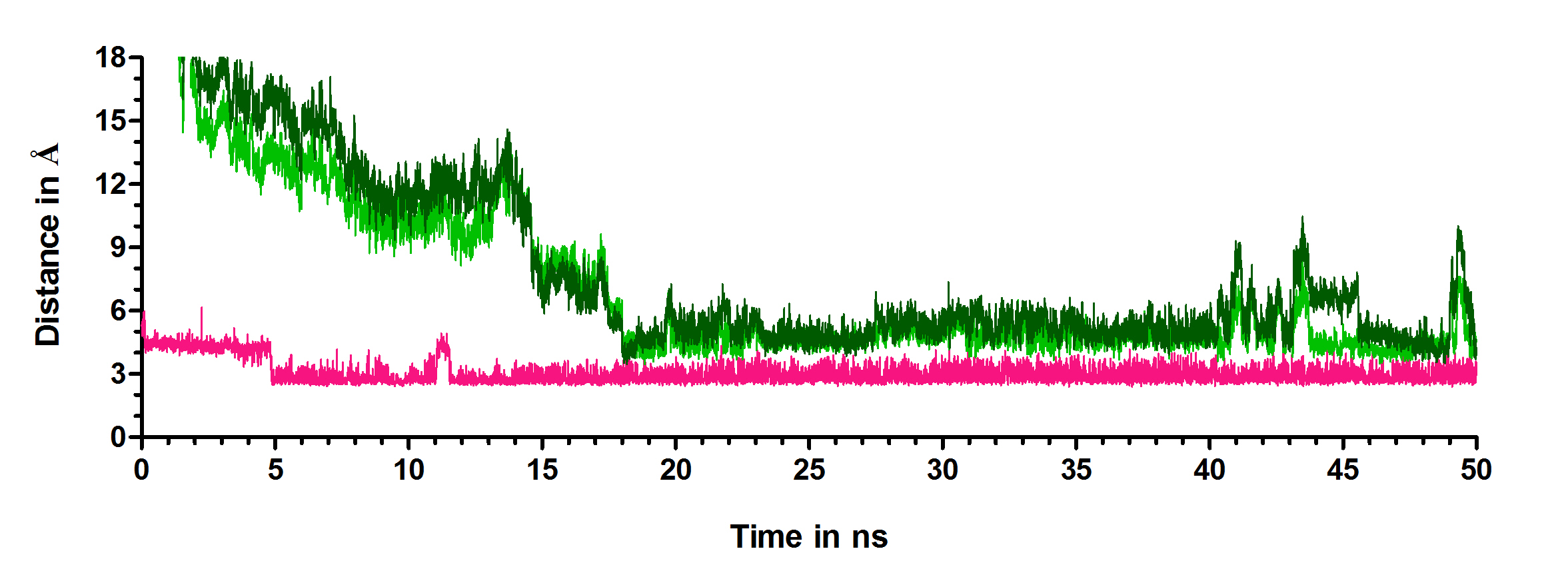


**Supplementary Figure S13.** **Entry of Mg^2+^ into the active site of monomer-A of Thr108Ser mutant DHBPS-Ru5P complex.** Figure shows distance between Mg^2+^ ion and O2 (dark green)/O3 (green) oxygen atoms of Ru5P. Ion enters the active site after the formation of a hydrogen bond between side chains of Thr-108’ and Asp114’. The bond is formed at ~ 5 ns. Distance between atoms in hydrogen bond is shown in pink.


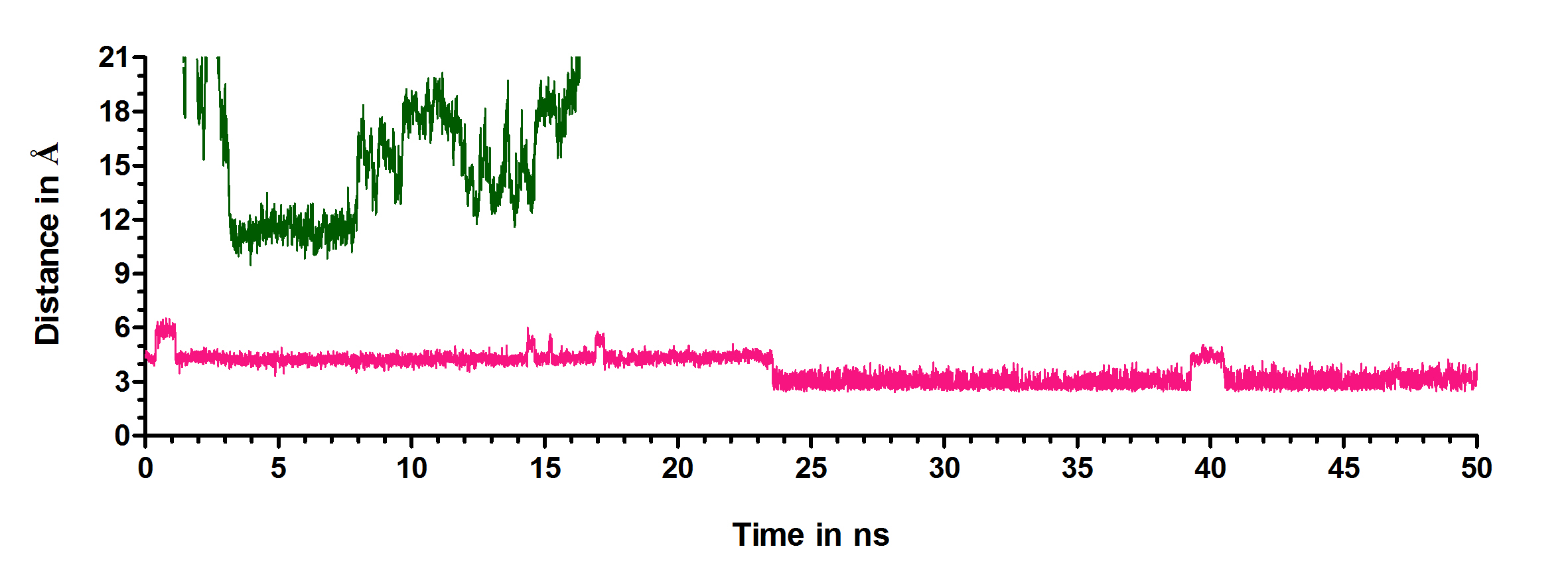


**Supplementary Figure S14**. **Entry of Mg^2+^ into the active site of monomer-B of Thr108Ser mutant DHBPS-Ru5P complex.** Figure shows distance between Mg^2+^ ion and O3 oxygen atom of Ru5P in dark green. Ion stays at a distance ~10-12 Å during 3 ns to 8 ns of simulation. However, it does not enter the active site as hydrogen bond between Ser-108 and Asp-114 is not formed as shown in pink.


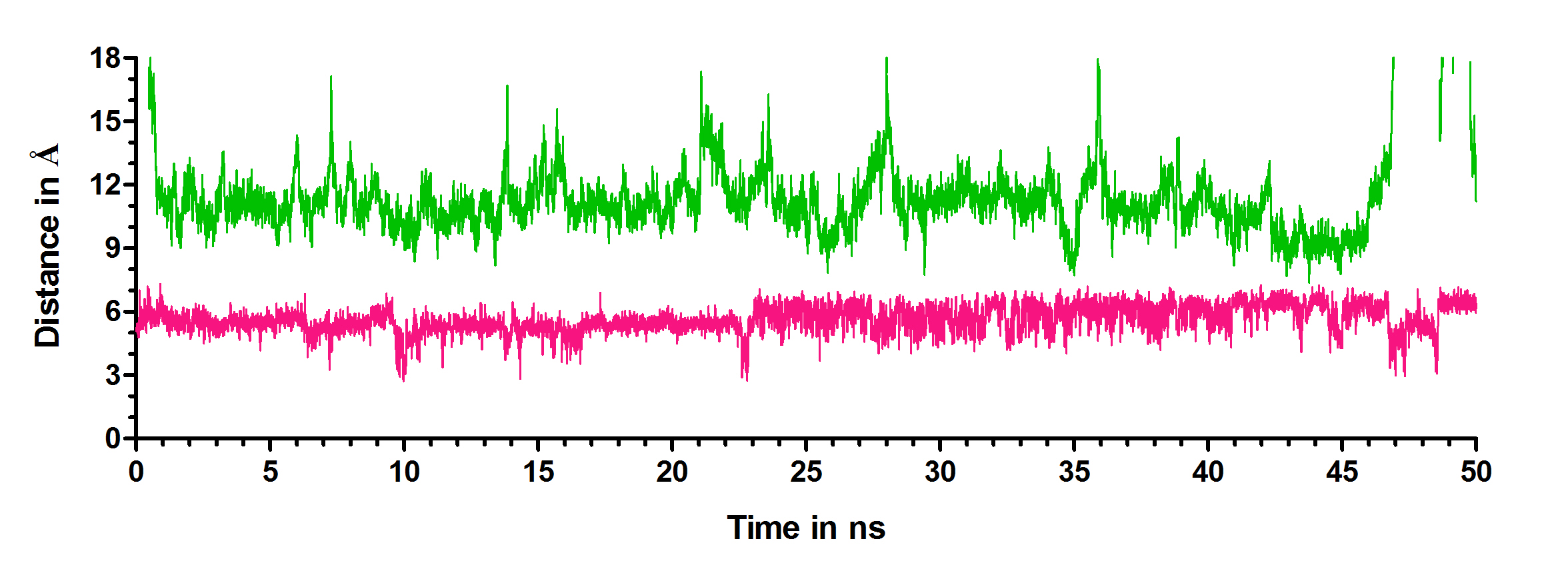


**Supplementary Figure S15.** **Entry of Mg^2+^ into the active site of monomer-A of Asp114Ser mutant DHBPS-Ru5P complex.** Figure shows distance between Mg^2+^ ion and O3 oxygen of Ru5P in green. During simulation ion stays at a distance ~11 Å from the Ru5P. However, it does not enter active site as hydrogen bond between Thr-108’ and Asp-114’ is not formed as shown in pink.


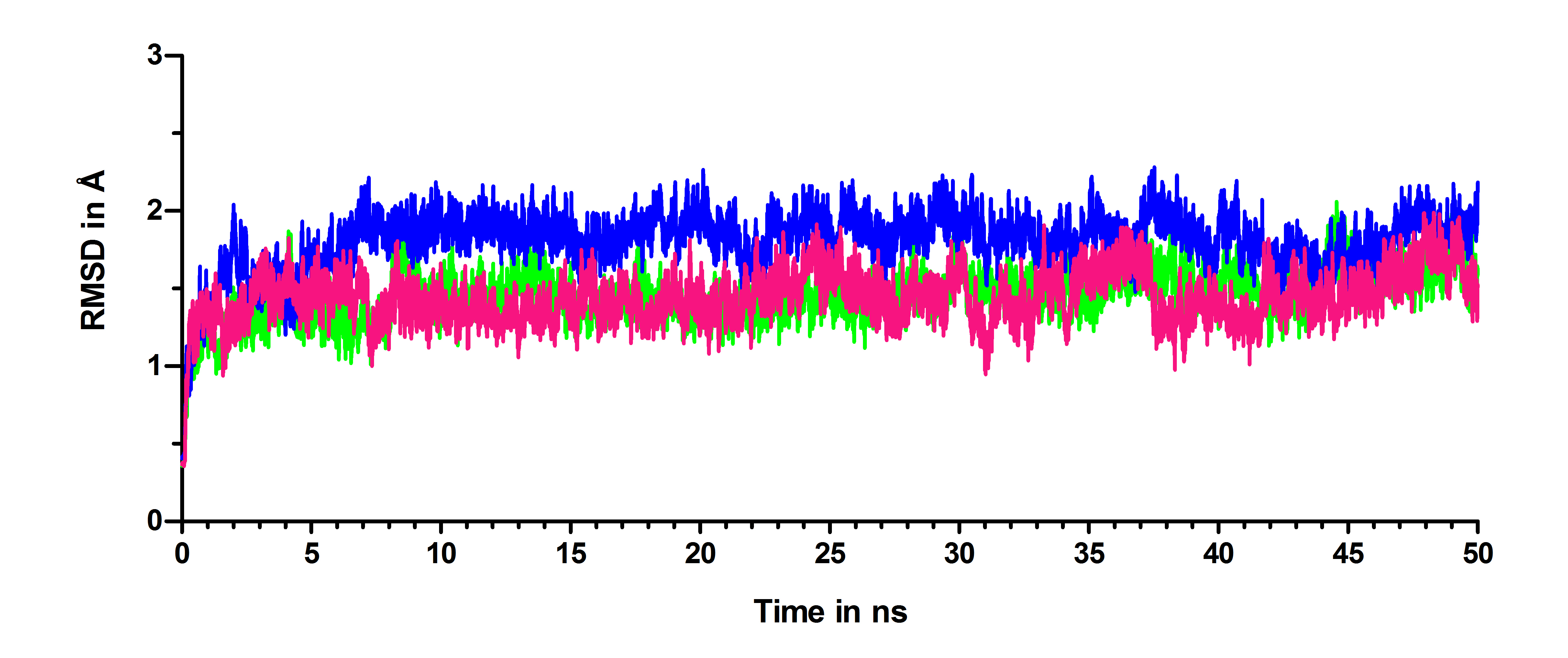


**Supplementary Figure S16. Stability of MD trajectories of inactive mutant complexes.** Root mean square deviation of backbone atoms during MD simulation of DHBPS-Ru5P complexes having a mutations- Glu39Ala, Glu41Ala, and His154Ala shown in green, pink, and blue, respectively.

**
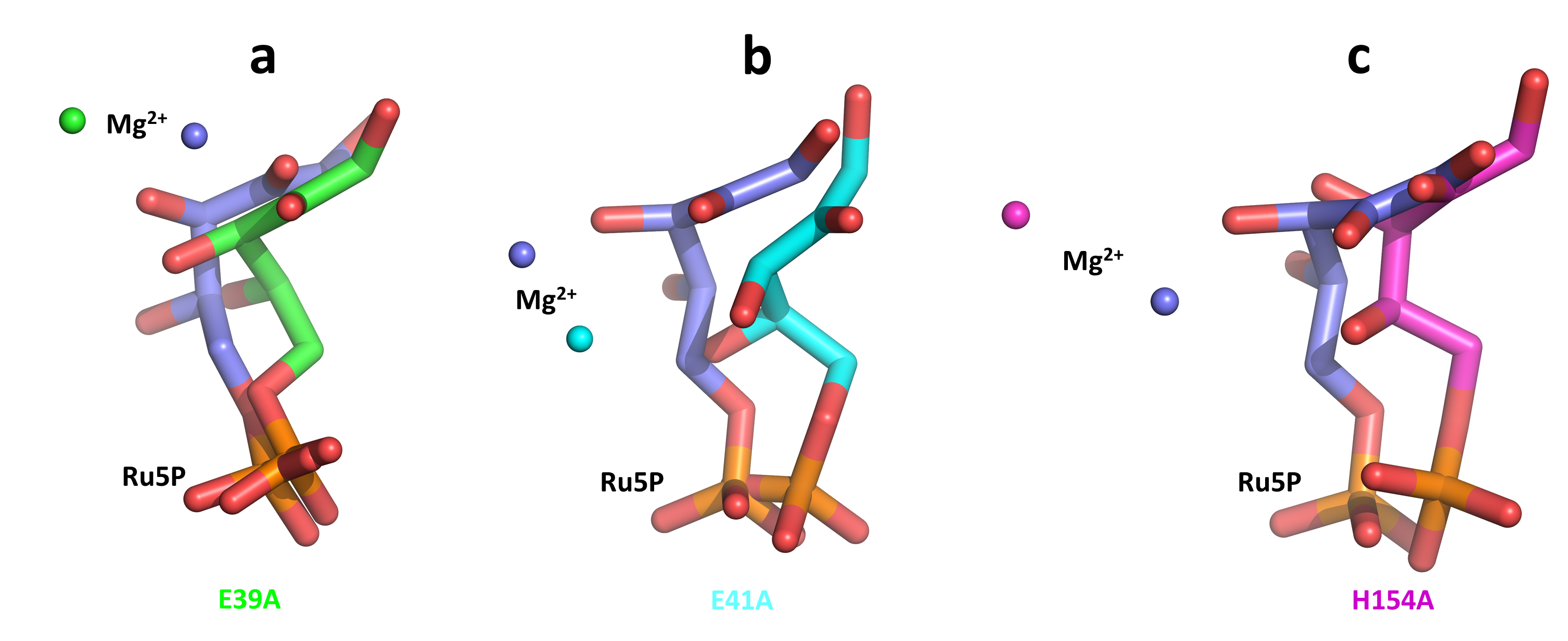
**

**Supplementary Figure S17. Conformations of Ru5P and positions of Mg^2+^ in the active sites of mutant and wild type complexes.** Figure shows superpositions of wild type DHBPS-Ru5P complexes (violet) with that of DHBPS-Ru5P complexes having a mutation of a. Glu39Ala (green), b. Glu41Ala (cyan), or c. His154Ala (magenta). 50^th^ ns conformation of DHBPS monomers that have shown the entry of Mg^2+^ are selected for the superposition.
